# Analyzing Overlaid Foreign Objects in Chest X-rays—Clinical Significance and Artificial Intelligence Tools

**DOI:** 10.3390/healthcare11030308

**Published:** 2023-01-19

**Authors:** Shotabdi Roy, KC Santosh

**Affiliations:** Applied AI Research Lab., Department of Computer Science, The University of South Dakota, Vermillion, SD 57069, USA

**Keywords:** chest X-ray, foreign objects, pulmonary abnormality, clinical significance, AI-guided tools

## Abstract

The presence of non-biomedical foreign objects (NBFO), such as coins, buttons and jewelry, and biomedical foreign objects (BFO), such as medical tubes and devices in chest X-rays (CXRs), make accurate interpretation difficult, as they do not indicate known biological abnormalities like excess fluids, tuberculosis (TB) or cysts. Such foreign objects need to be detected, localized, categorized as either NBFO or BFO, and removed from CXR or highlighted in CXR for effective abnormality analysis. Very specifically, NBFOs can adversely impact the process, as typical machine learning algorithms would consider these objects to be biological abnormalities producing false-positive cases. It holds true for BFOs in CXRs. This paper examines detailed discussions on numerous clinical reports in addition to computer-aided detection (CADe) with diagnosis (CADx) tools, where both shallow learning and deep learning algorithms are applied. Our discussion reflects the importance of accurately detecting, isolating, classifying, and either removing or highlighting NBFOs and BFOs in CXRs by taking 29 peer-reviewed research reports and articles into account.

## 1. Introduction

Chest X-rays are the most common, widely available, and affordable diagnostic imaging technique for cardiothoracic and pulmonary disorders [1]. Chest radiography is paramount in identifying abnormalities. CXRs are also used as an early diagnosis tool in the cardiothoracic region identifying lung and heart pathologies such as: atelectasis, consolidation, pneumothorax, pleural and pericardial effusion, cardiac hypertrophy, tuberculosis (TB), HIV and hyperinflation [1].

**Figure 1:**
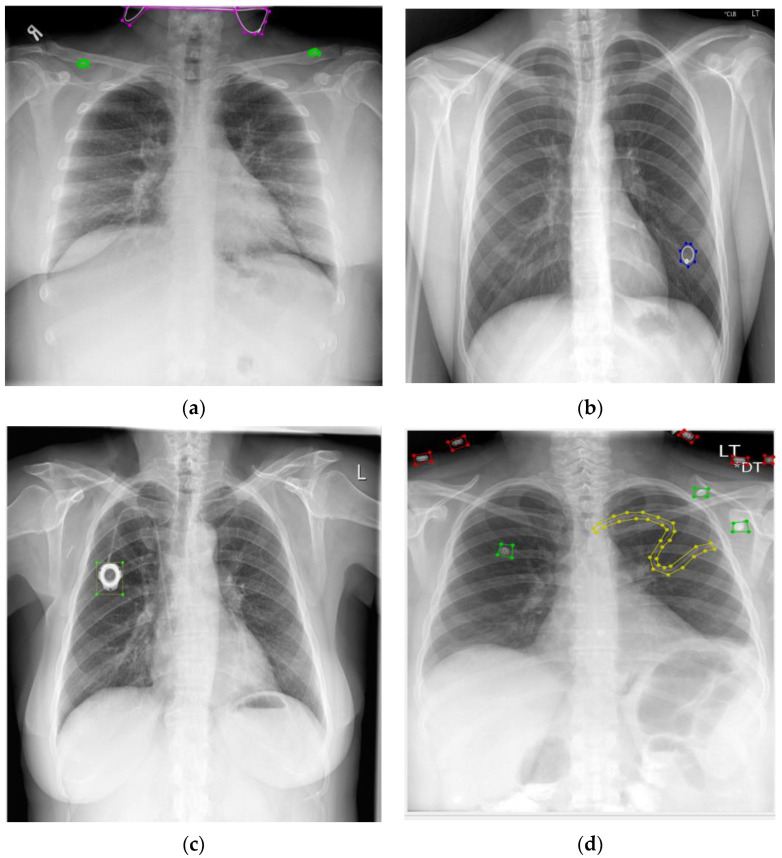
Foreign objects (annotated/labeled) in chest X-rays: (**a**) jewelry and buttons, (**b**) ring, (**c**) pacemaker, and (**d**) medical tube.

**Figure 2:**
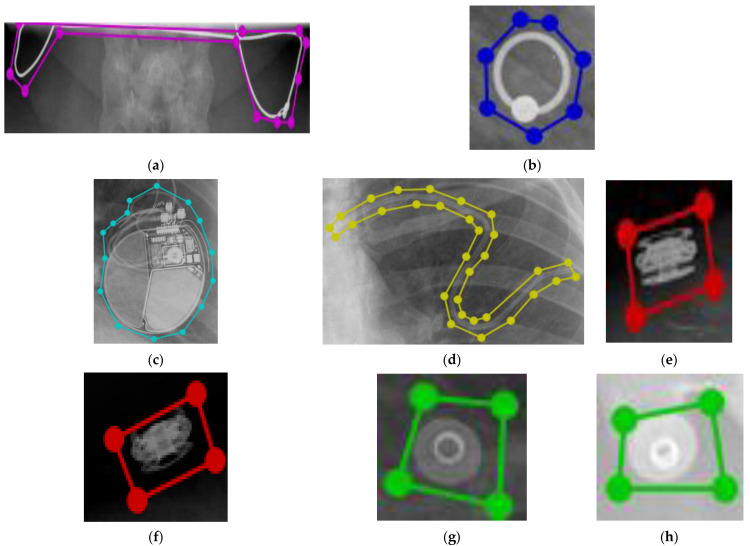
Closer view of NBFO: (**a**) chain, (**b**) ring; and BFO: (**c**) medical device, (**d**) tube, (**e**)–(**f**) buttons, (**g**)–(**h**) pinnode.

The detection of pulmonary abnormalities concerning tuberculosis (TB) with the presence of foreign objects could influence decision-making procedures [2]. An extensive number of individuals worldwide suffer from serious lung diseases, such as tuberculosis, pneumonia, lung cancer, and pulmonary edema [3] according to a World Health Organization (WHO) report. A total of 1.5 million people died from TB in 2020 (including 214,000 people with HIV). Worldwide, TB is the 13th leading cause of death and the second leading infectious killer after COVID-19 (above HIV/AIDS) [4]. A total of 1.80 million people has died from lung cancer [5] and pneumonia accounts for 14% of all deaths of children under 5 years old, killing 740,180 children in 2019 [6]. The development of CADs for automatic CXR screening has been prompted by the introduction of new, very effective hardware and software methodologies [7,8,9] (TB cases). More than 6.5 million people have died due to COVID-19 [10] which could have been less if we had considered the CAD system during the pandemic [11,12,13]. However, without proper instructions and training CAD can give us false-positive results. For example, foreign elements, such as buttons, coins, medical tubes, and medical devices within the CXR images hinder the performance of the automatic screening system, and we can divide these foreign objects into two groups (BFO and NBFO). Any medical devices, such as pacemakers, buttons, and pinnodes which can be found on a medical gown that patients are wearing, and medical tubes, jewelry, and so forth are considered as a BFO in CXR images. On the other hand, coins or rings which are mistakenly swallowed by patients, candy wrappers, broken toys, and so forth are considered NBFO in CXR images. BFO is a nonharmful foreign object and it is not considered a lung abnormality in CXR images [14,15]. For example, people who are in an intensive care unit (ICU) need BFO, or people who have a pacemaker in their heart cannot get rid of it in the CAD process. Meanwhile, if we failed to detect, localize, and identify NBFO early, it could be a serious threat to our health. Even clinicians could sometimes fail to detect it properly. For example, a tiny circle is considered an abnormality for TB. However, it could be buttons, rings, or coins. Therefore, in the screening process, precise detection of foreign objects is an important issue for screening chest diseases in CAD systems. Figure 1 shows the BFO (medical device, tube, buttons, pinnode) and NBFO (chain, ring) in CXR images with annotations. Figure 2 shows a closer view of the NBFO (chain and ring) and BFO (medical devices, medical tube, buttons, and pinnodes).

This paper focuses on the impact of NBFOs in CXRs by reviewing clinical studies and how much work has been done to detect and classify NBFOs and BFOs using artificial intelligence (AI)-guided tools. In Section 2, we describe our search criteria. In Section 3, we review clinical significance by considering NBFOs and their possible impacts. We then discuss the use of AI-guided tools to detect and localize foreign objects in CXRs. Section 4 includes both shallow learning and deep learning algorithms. While reviewing the performance of machine learning, we review data sets and their respective sources in Section 5. Section 6 concludes our study.

## 2. Inclusion/Exclusion Criteria

Our review process includes search keywords, search space, inclusion criteria, and exclusion criteria.

*Search keywords:* (chest X-ray OR chest radiograph) AND (foreign object detection) OR object detection.

*Search spaces:* PubMed and Web of Science.

As we primarily consider experimental papers with clinical significance that are primarily aimed for pulmonary abnormality screening, we reviewed 29 papers in total. We did not consider preprint papers (e.g., ArXiv, medRxiv, and TechRxiv) as they are not peer-reviewed.

## 3. Clinical Significance

In this section, we review the impact of BFOs and NBFOs from a clinical perspective, and based on literature from the 1980s onward, we consider the following types of NBFOs: magnets, button batteries, coins, disk magnets, scarf pins, metallic candy wrappers, as well as BFOs which turned out to be chest tubes, automatic implantable cardiovascular defibrillators, cardiopulmonary devices, pacemakers, catheters, intra-aortic counter pulsation balloon pumps, ventricular assist devices, endotracheal and tracheostomy tubes, nasogastric and nasoenteric tubes, and medically implanted wires, to name a few. The following discussion highlights these NBFOs and BFOs as they appear in CXRs.

Batteries and magnets are common in children’s toys and the ingestion of these items is an increasingly common theme with children. The harm in swallowing these items is often difficult to express for young children, including autistic, developmentally delayed, non-verbal, or non-disclosing children. In the Ref. [16] the authors showed a case study where challenges occurred in identifying and locating magnets and batteries in CXRs ingested by children. They reported that mild symptoms and signs resulted in a delayed diagnosis leading to serious consequences. Initially, these symptoms were mistakenly diagnosed as gastric issues, which prompted the CXRs. In the Ref. [17] Pugmire et al. presented a 15-year single-centered review from 2000 to 2015, based on clinical reports and images from Cincinnati Children’s Hospital Medical Center, where 276 cases of battery ingestion and insertions were confirmed. Fuentes et al. provided a case report in the Ref. [18] in which button batteries made up a small percentage of all foreign bodies eaten by children, and their position in the esophagus is even less common, making them more likely to cause serious injury. They presented three cases of children (2–7 years old) in which CXR imaging revealed circular NBFOs in the middle esophagus with unilateral esophageal burns (EB). In two of the three patients, the EBs progressed to esophageal stenosis. The researchers discovered 29 more cases in their investigation, with injuries including EB, esophageal perforation (EP), and tracheoesophageal fistula (TEF). They also stated that dysphagia or odynophagia were the primary symptoms in most patients (56%); however, non-specific symptoms, such as irritability, vomiting, and cough were also prevalent.

The growing number of small electronic devices with an electric supply by lithium button batteries (LBBs) has increased the number of cases of LBB ingestions, causing severe medical complications [19]. In the Ref. [20] Meyer et al. presented a study where the main aim was to analyze relevant diagnostic aspects of lithium button battery (LBB) X-ray imaging, and retrospective analysis of the imaging of radiopaque foreign bodies. For this study, they listed commercially available LBBs and alternate NBFOs, such as European coins (EC) and disk magnets (DM) according to their sizes and compositions. Thompson et al. compared digital and analog radiographs of the chest for use in detecting and evaluating a variety of cardiopulmonary devices (BFOs) in 40 patients which included 23 endotracheal tubes, 21 Swan–Ganz catheters, 14 central venous pressure catheters, 11 prosthetic valves, 10 chest tubes, six pacemaker wires, and five intra-aortic balloon pumps in the Ref. [21]. The validation of 40 digital and analog film pairs was compared by five radiologists, who assigned confidence levels for various judgments about each device. The results showed that there were no statistically significant differences in the identification of the devices except for prosthetic valves (all valves were detected on digital radiographs, compared with 62% on analog radiographs). The devices were detected on 96% of the digital radiographs and 90% of the analog radiographs.

The radiographic evaluation of the support and monitoring devices used in patients in the intensive care unit (ICU) is critically important because the potentially serious complications arising from their introduction and use are often not clinically apparent. For example, central venous catheters, pulmonary artery catheters, left atrial catheters, transvenous pacemakers, automatic implantable cardioverter defibrillators, intra-aortic counter pulsation balloon pumps, and ventricular assist devices are frequently used in critically ill patients. Godoy et al. [22,23] discussed and illustrated normal and aberrant positioning of support and monitoring devices frequently used in critically ill patients, including endotracheal and tracheostomy tubes, chest tubes, and nasogastric and nasoenteric tubes, as well as their inherent complications in radiography. Jennings et al. [24] compared computed and traditional radiography for the identification and visualization of cardiovascular devices in intensive care unit patients. Using a 2K × 2K, 12-bit storage phosphor plate technique that is readily available, they produced their calculated pictures. Three independent observers evaluated the validity of these 50 patient image sets. The observers noted a large variation in the sorts of images discovered for the detection of prosthetic valves and mediastinal drainage tubes. Greater assurance in identifying courses and line tips was seen in the computed pictures. They claimed that knowledge of typical and aberrant radiography findings is crucial for spotting these devices.

In the Ref. [25] Grier et al. reviewed the chest radiographs of 600 consecutive patients to review the necessity of chest radiography after undergoing insertion of a permanent cardiac pacemaker. The incidence and nature of CXR abnormalities were reviewed. They reported that abnormalities were detected on the chest radiographs of 131 patients (21.8%). Unsatisfactory electrode tip positions and other features related to the electrode wire were most common (14.4%). Complications related to the lungs and pleura were present at 5.5%, and those related to the generator and pouch were least frequent (1.9%). Complications occurred more frequently following the installation of a replacement system (48.3%) compared to new systems (17.2%). Important complications not initially detected included pneumothorax (8/15) and poor electrode loops (26/27). Chest radiographs following permanent cardiac pacing frequently demonstrate significant abnormalities whose detection is improved by awareness of their incidence and nature.

Murthy et al., in the Ref. [26] provided a case report of six patients who were brought with a history of aspirating a sharp foreign body (scarf pin). The ages of the patients, all of whom were female, ranged from 5 to 16. Between three hours and a day following the unintentional consumption, patients were admitted to hospitals. None of the patients had any localized respiratory obstruction symptoms or indications. They provided examples of the risks associated with mouth-holding by girls. It was demonstrated how to handle these pointed, potentially piercing foreign bodies. On the other hand, the majority of airway NBFOs occurs in children between the ages of 6 months and 6 years. Candy is something that children are particularly fond of, resulting in a high chance that kids may consume candy wrappers as well as candy. In the Ref. [27] Orgill et al. presented a case where the main goal was determining whether conventional and dual-energy radiographic techniques would exclude the presence of aspirated metallic foil wrappers. Single-layer and multi-layer metallic candy wrappers were radiographically studied with conventional and dual-energy radiographic techniques in three tissue models. They reported that no single-layer metallic samples were detectable with conventional or dual-energy radiography. The multilayer samples were non-detectable at less than 8 layers (pulmonary tissue model) or 16 layers (mediastinal model) by either conventional or dual-energy radiography. Findings indicate that conventional and dual-energy chest radiographic techniques do not reliably exclude the presence of aspirated metallic foil wrappers.

Coins currently represent the most retained esophageal foreign body among children in the United States [28,29,30]. Suspicion for coin ingestion is typically brought on by witnessed ingestion or symptoms such as gagging, choking, vomiting, and dysphagia [28]. In the Ref. [31] the focus of Huyett et al. was to determine the accuracy of CXRs in children using ingested radiopaque NBFOs of known size. A database of foreign body ingestion at a tertiary care children’s hospital was queried from 2013 to 2016 for children who had ingested US coins, had a pre-operative chest X-ray, and documentation of coin type at the time of endoscopic removal. Four blind research tests measured the coin diameter on chest X-rays using iSite PACS software, and based on the measurement, predicted the coin type. Researchers concluded that the measurement of esophageal coins on chest X-ray was relatively inaccurate and overestimates the size in most cases, and clinicians should exercise caution when performing fine measurements on chest X-rays, especially in children younger than 4 years old. Raney et al., in the Ref. [32] presented a case study where a child had ingested an esophageal coin, but radiographic findings supported a coin located in the trachea. This case study illustrates the importance of performing radiographic studies that include both anteroposterior and lateral neck/chest views in patients who aspirate or swallow coins. The purpose of this report is to show that there are exceptions to the guidelines used to interpret roentgenograms for the determination of esophageal or tracheal coin location. Tander et al. evaluated patients with coins retained in the esophagus and the impact of the size of the coins on lodgment location in the Ref. [33]. They investigated 62 children with a history of coin ingestion and a chest X-ray, showing a retained coin in the esophagus. They reported that there were 27 male and 35 female patients with coin lodgment (median age, 4 years; range, 1–13). Forty-five patients (73%) ingested a coin with a diameter between 23.45 and 26.00 mm. In the remaining 17 patients (27%), ingested coins had a diameter between 17.00 and 20.90 mm or between 26.85 and 28.00 mm. Fifty coins were localized in the upper esophagus, eight coins were found in the middle esophagus, and four patients had a coin in the distal esophagus. There was a positive correlation between the diameter of the coin and the age of the patient (r = 0.415 and P¡ 0.001). Even though esophageal NBFOs in children remains an important and complex medical subject, these accidental ingestions can be dangerous for adults as well. Teenagers often keep pen tops or pins in their mouths during their routine daily activities. In the Ref. [34] Rybojad et al. worked with a study of 192 cases of suspected esophageal NBFOs between 1998 and 2010. One radiopaque showed a wedding ring in the esophageal area. Rybojad et al. mentioned that sometimes NBFO ingestions can be difficult to diagnose. In suspected cases, the doctors X-rayed both a lateral chest and neck profile. The authors discussed clinical symptoms and radiological findings of variable esophageal NBFOs as well as therapeutic procedures in pediatric patients. Data were statistically analyzed by a chi-square test. Reporting’s demonstrated that a NBFO was removed from the digestive tract of 163 children aged 6 months to 15 years (mean age 4.9). Most objects were located within the cricopharyngeal sphincter. Dysphagia occurred in 43%, followed by vomiting (29%) and drooling (28%). The most common ingested objects were coins. Ordinal chest X-rays demonstrated aberrations in 132 cases, and in uncertain situations, an esophagram test was additionally ordered. For the group of 37 patients whose radiograms were normal, esophagoscopy revealed fifteen more NBFOs, which were eventually successfully removed. In the Ref. [35] Schlesinger and Crowe evaluated the clinical presentation and radiographic appearance in eight cases of esophageal coins in children with an atypical sagittal orientation on chest radiographs. They reviewed patient age, sex, type of coin, location of the coin within the esophagus, method of coin removal, presence of underlying esophageal anomalies, treatment, and complications related to the coin ingestion or removal among the clinical records and chest radiographs of eight children. They reported that the age of the eight children ranged from 3.8 to 17.7 years (mean, 7.8 years), the coins were lodged at the level of the aortic arch in seven of the eight patients and the level of the distal third of the esophagus in one patient. One of the eight cases showed the coin was originally in the sagittal plane but spontaneously reoriented into the coronal plane. They also mentioned that the coins with a sagittal orientation on chest radiographs in the trachea were usually not correct. A coin seen with a sagittal orientation on a chest radiograph will more likely be in the esophagus.

In the Ref. [36] Ullal et al. presented the case study of a new investigative technique of virtual bronchoscopy, which is useful in locating non-radiopaque NBFOs missed using ordinal radiography. The aim was to study the clinical profile of patients with suspected NBFO aspiration and to evaluate the changing trends in the diagnosis for quicker management of NBFO aspiration by the way of virtual bronchoscopy. For validation, Ullal et al. reviewed the medical records of patients with NBFO aspiration from August 2006 to September 2016. They reported that in 150 patients with NBFO aspiration, detected by virtual bronchoscopy, 148 patients were diagnosed to have NBFO by rigid bronchoscopy. This amounts to a positive predictive value of 97.3% which was like the positive predictive value of rigid bronchoscopy at 99%. Virtual bronchoscopy is the only imaging modality that returned 99.9% reassurance about the presence or absence of a NBFO, because of both its high sensitivity and specificity, proving to be a lifesaving tool. On the other hand, Morrier et al. [37] evaluated the efficacy of a seed-migration detector and compared its performance to fluoroscopy and postoperative chest radiographs. Their propositions use a gamma scintillation survey meter which was converted to a seed-migration detector by adding a shield on the probe detection window. To validate, the detector was used to perform a chest evaluation on 737 patients at their first postoperative visit. When the detector showed positive activity, seed migration was confirmed by taking a chest radiograph and by looking at the region with fluoroscopy. They reported that 103 patients (14.0%) presented at least one embolized seed. This accounts for 123 of the 39,887 seeds. A total of 87, 12, and 4 patients had 1, 2, and 3 seed embolizations, respectively. Compared with the seed-migration detector, detection based on fluoroscopy had led to 13 false-negative detections (of 103, or 12.6%), and the radiograph had resulted in 31 or 30.1. In the Ref. [38] Kero et al. presented a report during the years 1969–1981 where 57 children with inhaled NBFOs in the tracheobronchial tree were treated at Turku University Hospital. They reported that 91% had a history of NBFO inhalation and 25% had a radiopaque NBFO which was seen in the CXR. A total of 9% of 57 patients had a NBFO discovered unexpectedly through bronchoscopy. These NBFOs were removed by bronchoscopy from all the patients but one, who required a segmentectomy due to a fragment of a spike in the lung parenchyma. Overall, we found that it is frequently challenging to identify NBFOs in clinical settings since patients rarely exhibit any specific symptoms, even though the longer they remain in the chest region, the greater the risk to their health. Additionally, employing CXR to detect NBFOs early in clinical fields is not an effective method, which motivates us to examine CAD performance, as detailed in the following section of our research. In Table 1, all these cases and subjects are described from clinical observation.

## 4. AI-Guided Tools for NBFO and BFO

**Figure 3:**
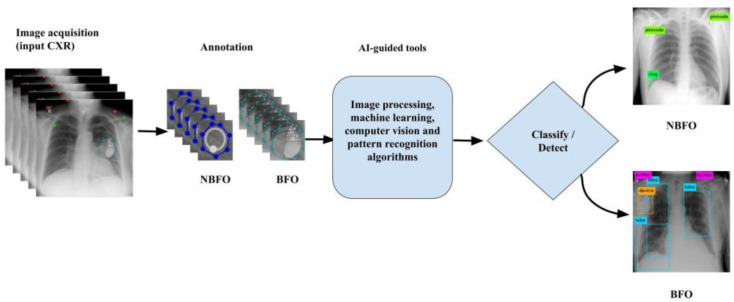
Typical workflow of the AI-guided tools for foreign object (NBFO and BFO) classification/detection.

Typical AI-guided tools work as demonstrated in Figure 3. In Figure 3, annotated NBFOs and BFOs are used to train AI-guided tools that employ image processing, machine learning, computer vision, and pattern recognition algorithms to detect/classify foreign objects: NBFO and BFO.

Automatically detecting foreign objects in CXRs is not trivial [39] and it does not have rich state-of-the-art literature. Often, shallow learning algorithms are used, and feature engineering covers most of it, where basic image processing and pattern recognition techniques are common. A technique to identify NBFOs in chest X-rays, such as buttons on the patient’s robe, was developed by Zohora et al. [2]. The four main techniques used in these claims—intensity normalization, low-contrast picture identification and enhancement, segmentation of lung areas, and button object extraction are based on the Hough transform and the Viola–Jones algorithm. Using a ground truth dataset of 505 button objects, they examined and contrasted both methods for validating these approaches. Using a new method, Zohora et al. [40] mentioned the detection of circular NBFOs and BFOs (such as buttons and medical devices) in lung areas. They first created an edge map using a variety of edge detection methods, then performed morphological procedures to choose possible candidates. They applied the circular Hough transform (CHT), and with lung segmentation, they reported precision, recall, and F1 scores of 96%, 90%, and 92%, respectively. Their results are comparable with state-of-the-art works. An automated approach was created by Hogeweg et al. [41] to identify and eliminate NBFOs and BFOs from chest radiographs, such as buttons, brassier clips, jewelry, pacemakers, and wires. These methods produced a probability estimate for each pixel belonging to a projected NBFO and BFO by using supervised pixel classification using a kNN classifier. By grouping and post-processing pixels with a probability greater than a specific benchmark threshold, segmentation was carried out. In paintings, texture took the place of actual objects. They assessed trials on 257 chest radiographs and the reported accuracy of the detection at a pixel level value of 0.949 to validate this method. At the object level, free response operator characteristic analysis revealed that 95.6% of objects were recognized with an average of 0.25 false-positive detections per image.

Schultheiss et al. [42] developed a design to improve the early detection of lung cancer in computed tomography and CXR. Based on segmented pulmonary nodules, their model trained a convolutional neural network (CNN) based on a single-stage detector (RatinaNet) with 257 annotated radiographs and 154 additional radiographs from a public dataset. They conducted a reader study with 75 cases to validate the design and found that for nodule location detection, the architecture had a performance of 43 true-positives, 26 false-positives, and 22 false-negatives. They also compared the performance of the convolutional neural network with the performance of two radiologists. In contrast, the dual readers performed with 42 true-positives and 2 false-positives, 28 true-positives and 0 false-positives, and 23 false-negatives. They found a ROC, AUC value of 0.87 for the reader study testing the trained RetinaNet architecture to be only marginally susceptible to detect NBFOs and BFOs in terms of misclassifications: out of 59 additional radiographs containing NBFOs and BFOs, two radiographs had false positives that were mistakenly identified as foreign bodies.

Deshpande et al. [43] proposed a design to classify NBFOs (e.g., tubes and wires, pacemakers, implants, small external objects, jewelry, and pushbuttons) in CXRs. They used a deep learning framework with the subset of the MIMIC CXR dataset and annotated 6061 images with the primary purpose of classifying NBFOs. Their networks were pre-trained using ImageNet on the NIH database ChestX-ray14. Using five-fold cross-validation on 4704 images plus an additional test set containing 1357 images, they reported classification AUCs of 0.984 (for binary classification) and 0.96 (for multilabel classification). Note that their objective was to classify foreign objects, not detection and/or localization of foreign objects. As a result, it was not included in Table 2.

Santosh et al. [44] created a method that used a faster region-based convolutional neural network to detect circle-like NBFOs (such as coins and buttons) of various sizes during an automated CXR screening process (R-CNN). On a set of 400 publicly accessible CXR images hosted by LHNCBC, the U.S. National Library of Medicine (NLM), and the National Institute of Health (NIH), the authors validated the use of the proposed deep neural network (DNN) and achieved 97% precision, 90% recall, and a 93% F1-score. All these reported works were mostly limited to circle-like foreign object detection. Using this same dataset, Santosh et al. [45] published their recent work based on a YOLOv4-a DNN-based object detection technique, and they were able to detect all kinds of foreign objects (BFO and NBFO) in their research. They achieved the following performance scores: accuracy of 91.00%, precision of 85.00%, recall of 93.00%, and f1-score of 89.00%. Unlike state-of-the-art works, where they are limited to a specific type of foreign object (e.g., circle-like objects), this is the first time they reported experimental results on all possible types of foreign objects. In Table 2, the methods and performance of detecting BFOs and NBFOs are described.

## 5. Data Description

In this section, we summarize datasets used in Section 4. Table 3 includes the dataset name size and the author’s name, which are mentioned in Section 4. The National Library of Medicine (NLM) dataset, which had been preserving a sizable dataset of chest radiographs as well as related radiological reports, was used by Xue et al. [39]. This dataset, compiled by the medical school at the University of Indiana, includes around 4000 frontal and lateral chest X-ray DICOM image pairings and 4000 textual reports that correlate to the images [46]. It was incorporated into Open-i, a multi-modal system for retrieving biological literature created by NLM [47]. The creation and evaluation of computer-aided diagnosis (CAD) algorithms for lung disorders can benefit from this dataset.

Zohora et al. [2,40] used a subset of the dataset maintained by the National Library of Medicine (NLM) composed of 50 DICOM and 400 DICOM CXRs images. There was a total of 32 buttons on the chest of this subgroup of the data set. Since they are not present in this data set, they marked the related ground truths. Additionally, Santosh et al. [44,45] used the same dataset of 400 CXR images in their experimental work.

For evaluation of the detection and removal of NBFO, Hogeweg, et al. [41] used a large chest radiograph database of 257 chest radiographs from a tuberculosis screening program for high-risk groups in London [48]. Each image was captured digitally using a single device (DigitalDiagnost Trixel, Philips Healthcare, the Netherlands). According to the size of the source image, all photos in this work were scaled to a width of 1024 pixels, giving them resolutions ranging from 0.22 to 0.38 mm per pixel. They discovered that this resolution is adequate for the detection of tuberculosis-related anomalies in chest radiographs, even though the original resolution is about a factor of 2 higher (0.144 mm pixel size). For the identification and segmentation of NBFOs, this resolution also proved sufficient. Full-resolution photographs could also use inpainting if desired. NBFOs can be found in this database’s photos in about 20% of the cases. A subset of this database was used to evaluate the automatic detection of foreign objects and their effect on the automatic detection of textural abnormalities.

A dataset of 391 CXRs was collected from Schultheiss et al. [42] and the institution’s picture archiving and communication system (PACS). Therefore, case-level ground truth labels (unsuspicious or nodulous) were assigned based on the diagnosis of two radiologists: the first radiologist made the diagnosis according to a clinical routine, and a second radiologist (who had 3 years of experience in chest imaging) verified and segmented the nodules, retrospectively, using our in-house built web-based platform. Another radiologist checked the segmented nodules for the reader study test set (12 years of experience). Based on the segmentation boundaries, bounding boxes were extracted from the segmentations. A total of 257 radiographs with nodules were utilized for training purposes. The Japanese Society of Radiological Technology (JSRT) dataset 31, from which 154 additional radiographs with labeled nodules were obtained, was used to complement the training data. Consequently, 411 radiographs were used in total for training. To train a lung segmentation network, lung segmentations for each of the 247 JSRT files were also taken from the segmentation in chest radiography (SCR) database 32. Please take note that 93 more non-nodulous photos from the JSRT database were included in the data for lung segmentation. For the lung segmentation train, validation and test set sizes were set to 157, 40, and 50.

## 6. Conclusions

In this paper, we analyzed overlaid foreign objects in chest X-ray (CXR) images by considering two factors: clinical significance and AI-guided tools. Both non-biomedical foreign objects (NBFOs) and biomedical foreign objects (BFOs) were considered in our study since we were required to have effective reading and accurate interpretation of CXRs. While reviewing, we observed that NBFOs range from accidentally ingested batteries, coins, hair pins, jewelry, candy wrappers, hardware, or toys, and BFOs include biomedical devices like pacemakers, medical device wiring, medically installed prostheses, and medical tubing. In addition to clinical significance, we examined the detailed discussions on numerous clinical reports in addition to computer-aided detection (CADe) with diagnosis (CADx) tools. In CADe and CADx, both shallow learning and deep learning algorithms were considered. When it comes to automation, AI-guided tools (with the possibility of having explainability and/or interpretability) are a must, but they require larger datasets.

## Figures and Tables

**Table 1:** Foreign objects in chest X-rays (clinical observation) dataset (cases and subjects).

Authors	Foreign Objects	Method	Dataset (Case) (Subjects)
Brown et al. (2012) [16]	Magnets and batteries	Case Study	1 Subject
Pugmire et al. (2016) [17]	Button Battery	Review	276 cases
Fuentes et al. (2014) [18]	Button Battery	Review and Case Study	3 Cases and 29 Review
Meyer et al. (2020)) [20]	Button Battery, Coins, Disk magnets	Case Study	20 subjects
Thompson et al. (1989) [21]	Cardiopulmonary devices	Digital and Analog	40 subjects
Godoy et al. (2012) [22]	Catheters, Pacemakers, Automatic implantable cardioverter defibrillators, intra-aortic counter pulsation balloon pump, ventricular assist devices	Case Study	-
Godoy et al. (2012) [23]	Endotracheal and tracheostomy tubes, Chest tubes, and Nasogastric and Nasoenteric tubes	Case Study	-
Jennings et al. (1992) [24]	Cardiopulmonary devices	Phosphor plate system	50 subjects
Grier et al. (1990) [25]	Pacemaker	Case Study	600 subjects
Murthy et al. (2001) [26]	Scarf pin	Case Study	6 subjects
Orgill et al. (2018) [27]	Multi-layer metallic candy wrappers	Duel-energy radio-graph	1 subject
Huyett et al. (2018)) [31]	Coins	Case Study	4 subjects
Raney, Losek (2007)) [32]	Coins	Case Study	1 subject
Schlesinger, Crowe (2011) [35]	Coins	Case Study	8 subjects
Tander et al. (2009) [33]	Coins	Case Study	62 subjects
Ullal et al. (2018) [36] Morrier et al. (2010) [37]	Foreign body	Case Study	150 subjects
	Seed-migration	A gamma scintillation	737 Subjects
Kero et al. (1983) [38] Rybojad et al. (2012) [34]	Foreign bodies	Case Study	57 subjects
	Esophageal foreign bodies	Chi-square test	192 subjects

**Table 2:** NBFO and BFO in chest X-ray, dataset size, and performance measured in Performance, Recall, F1-score.

Authors	NBFO, BFO	Methods	Datasets (Size of Images)	Performance (in %)		
				Precision	Recall	F1-Score
Xue et al. (2015) [39]	Buttons	Hand-crafted features	505	0.84	0.88	-
Zohora, Santosh (2017) [2]	Buttons, medical devices	Hand-crafted features	50	100	100	-
Zohora, Santosh (2017) [40]	Circular (buttons, medical devices)	Hand-crafted features	400	0.96	0.90	0.92
Hogeweg et al. (2013) [41]	Buttons, brassier clips, jewelry, or pacemakers and wires	kNN classifier	257	0.949 (pixel level value)	-	-
Schulthesiss et al. [42]	Nodule detection	CNN (RatinaNet)	411	-	0.87 (ROC)	-
Santosh et al. (2020) [44]	Circle like (e.g., coins/buttons)	R-CNN	400	0.97	0.90	0.93
Santosh et al. (2022) [45]	Buttons, coins, ring, pinnode, medical devices, tube	YOLOv4	400	0.85	0.93	0.89

**Table 3:** Datasets.

Dataset	Size	Authors
US NLM, National Institute of Health (Indiana Dataset)	278	Xue et al. (2015) [39]
US NLM, National Institute of Health	50	Zohora, Santosh (2017) [2]
US NLM, National Institute of Health	400	Zohora, Santosh (2017) [40] Santosh et al. (2020,2022) [44,45]
Digital Diagnost Trixel, Philips Healthcare, the Netherlands	257	Hogeweg et al. (2013) [41]
Japanese Society of Radiological Technology (JSRT)	411	Schulthesiss et al. [42]

## Data Availability

Not applicable.
